# Risk-Aware Identification of Highly Suspected COVID-19 Cases in Social IoT: A Joint Graph Theory and Reinforcement Learning Approach

**DOI:** 10.1109/ACCESS.2020.3003750

**Published:** 2020-06-19

**Authors:** Bowen Wang, Yanjing Sun, Trung Q. Duong, Long D. Nguyen, Lajos Hanzo

**Affiliations:** 1 Xuzhou Engineering Research Center of Intelligent Industry Safety and Emergency CollaborationXuzhou221116China; 2 School of Electronics, Electrical Engineering, and Computer ScienceQueen’s University Belfast 1596 BelfastBT7 1NNU.K.; 3 Duy Tan University 374802 Da Nang810000Vietnam; 4 School of Electronics and Computer ScienceUniversity of Southampton 7423 SouthamptonSO17 1BJU.K.

**Keywords:** Social Internet of Thing (SIoT), COVID-19, reinforcement learning, graph theory

## Abstract

The recent outbreak of the coronavirus disease 2019 (COVID-19) has rapidly become a pandemic, which calls for prompt action in identifying suspected cases at an early stage through risk prediction. To suppress its further spread, we exploit the social relationships between mobile devices in the Social Internet of Things (SIoT) to help control its propagation by allocating the limited protective resources to the influential so-called high-degree individuals to stem the tide of precipitated spreading. By exploiting the so-called differential contact intensity and the infectious rate in susceptible-exposed-infected-removed (SEIR) epidemic model, the resultant optimization problem can be transformed into the minimum weight vertex cover (MWVC) problem of graph theory. To solve this problem in a high-dynamic random network topology, we propose an adaptive scheme by relying on the graph embedding technique during the state representation and reinforcement learning in the training phase. By relying on a pair of real-life datasets, the results demonstrate that our scheme can beneficially reduce the epidemiological reproduction rate of the infection. This technique has the potential of assisting in the early identification of COVID-19 cases.

## I. Introduction

The coronavirus disease 2019 (COVID-19) has spread over 215 countries with the numbers of infected cases and deaths still increasing. As of the 19th April 2020, a cumulative total of 2,228,455 (154,309) cases (fatalities) were reported in the world. During this outbreak, every aspect of our daily lives has been deeply impacted. One of the gravest challenges is its high human-to-human transmission rate via droplet inhalation or contact with contaminated surfaces. Recent studies have demonstrated that asymptomatic patients are particularly contagious [1], [2], because people tend to avoid contact with others showing obvious symptoms, but asymptomatic people cannot be readily identified. Therefore, early identification of suspected cases and the judicious allocation of limited medical resources is vital [3].

Recently, both ‘big data’ analysis and human social networking solutions were proposed for detecting suspected cases during an epidemic. For example, in [4], the authors proposed a spatio-temporal model termed as HiRES, which relies on a risk map for detecting suspected individuals based on the trajectory of big data and mean-field theory. In [5], the authors proposed a sentinel node detection strategy for disease surveillance by relying on social networks. However, the latency in the associated trajectory and inaccuracy of social data may render these models somewhat inefficient. Hence, a deep-routed research-question arises, namely how to take advantage of both the real-time social data and of accurate trajectory data for identifying suspected virus careers. In [6], the authors proposed the Social Internet of Things (SIoT) concept, which paves a new way for building the social relationships among devices without human intervention. Despite the delay in human data feedback, portable equipment such as smart phones and wearable devices may be employed for sensing, computation and communications, while relying on positioning information to perform real-time symptom recognition, contact tracking and data exchange. For example, the so-called co-located object relationship (C-LOR) of the SIoT characterizes the geographic location similarity of two devices, while the social object relationship (SOR) quantifies the contact intensity, when the device-owners are in each others’ proximity, which are useful for identifying the individuals at risk of infection.

By collecting the data from SIoT, the complex networks of virus transmission may be viewed as a weighted undirectional graph (WUG), where each vertex represents a mobile user, each edge indicates the contact between two users and the vertex weight is related to the probability of becoming infected. Based on this graph, we will identify those vertices which may have high impact on other vertices, corresponding to the influential individuals in a resource-constrained environment, since the medical resources such as surgical masks and nucleic acid detection reagents are scarce. Hence, the optimization objective is to select high-risk vertices within a limited budget of resources to minimize the propagation rate of the epidemic. This epidemic propagation rate minimization problem of identifying the suspected COVID-19 cases in SIoT may be viewed to be analogous to the rumor influence minimization problem of identifying the highly influential nodes in mobile social networks. The latter can be further transformed into the classic minimum-weight vertex cover (MWVC) problem of graph theory [7]. Most prior studies resorted to heuristic algorithms or to local search for solving MWVC problems at an acceptable complexity [8]. However, considering the dynamically evolving network topology over time, recomputing the solution from scratch is time-consuming. As an efficient decision-making technique in dynamic environments, reinforcement learning has been widely used in the field of wireless communications, aerospace, power system, etc [9], [10]. In [11], the authors proposed an adaptive strategy based on graph embedding and reinforcement learning for solving the associated combinatorial optimization problem, which inspired us to design an adaptive identification scheme for highly suspected COVID-19 cases in response to these topology changes.

The main contributions of this paper are summarized as follows:

• By using the dynamic WUG model, we propose a new network topology of SIoT-aided inter-device social relationship establishment process, which takes into account the fact that the network structure evolves dynamically throughout the epidemic propagation.
• We conceive the high-risk vertex selection problem relying on the MWVC framework and propose a risk-aware adaptive identification algorithm based on joint graph embedding and reinforcement learning for solving the MWVC problem in a dynamic topology.
• We conduct simulations based on a pair of realistic datasets to demonstrate that our proposed scheme is efficient in suppressing the propagation speed in both large-scale and small-scale scenarios. Besides, we evaluate our proposed scheme on the Erdos-Renyi social graph relying on adjustable contact probability to verify the scalability.

The rest of this paper is outlined as follows. First our system model is presented and then our optimization problem is formulated in Section II. The adaptive scheme for identifying the suspected cased with high risk is illustrated in Section III. Simulation results are shown in Section IV, followed by concluding remarks in Section V.

## II. System Model and Problem Formulation

In the absence of global restrictions COVID-19 spreads without limits since many asymptomatic carriers are contagious. To characterize the spread of COVID-19, we consider the modified susceptible-exposed-infected-removed (SEIR) epidemic model, where asymptomatic individuals are based on [12]. When a susceptible individual comes in contact with either a symptomatic or asymptomatic individual, the probability of being exposed is $\beta _{s} $ or $\beta _{a} $, respectively. Furthermore, the probability of those exposed individuals being symptomatic or asymptomatic is $\alpha _{s} $ or $1-\alpha _{s} $, respectively. Finally, the probability of being removed from the set through recovery or death is $\gamma $.

**FIGURE 1.**
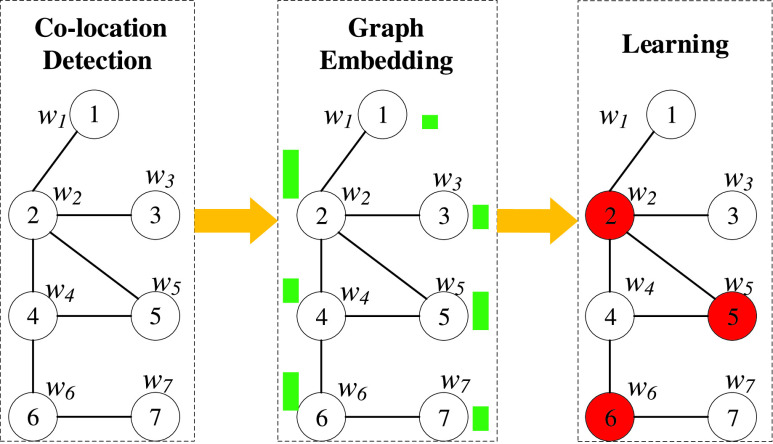
Framework of our proposed scheme for MWVC.

Naturally, the rate of propagation is also influenced by contact intensity, as determined by the contact frequency and duration. As shown in Fig. 1, each device can rely on the global positioning system (GPS), wireless network signaling, human social networks, radio frequency identification (RFID), bluetooth, and Wifi to track their owner’s contacts, perform co-location detection, and establish relationship with other devices through owner control and relationship management modules [6]. Then, the collected data will be gathered and aggregated by mobile vehicles or unmanned aerial vehicles, and finally delivered to the edge data center for real-time data analysis and decision-making. Herein, we mainly focus on the decision-making process since the data collection and analysis problems are beyond the scope of this paper and can be addressed by several existing works [3], [4]. Given a time span $\mathcal {T}$ which is discretized into $\{1,\ldots,t,\ldots,T\}$ time slots, the average contact intensity between two users $i $ and $j $ can be denoted as [13]

\begin{equation*} \overline \delta _{i,j}(t)=\dfrac {\sum _{x=1}^{N_{i,j}^{c}(t)}t_{x}^{d}}{N_{i,j}^{c}(t)},\tag{1}\end{equation*}

where $N_{i,j}^{c}(t) $ represents the total number of contacts before the time slot $t $, while $t_{x}^{d} $ is the corresponding contact duration. Note that even short exposures such as two seconds of contact are perilous and multiple short contacts do increase the overall risk of exposure. Furthermore, the weight of each contact is quantified by Eq. (1), where a short exposure time corresponds to a low weight.

Given an individual set $V(t)=\{v_{1}(t),\ldots,v_{i}(t),\ldots,v_{N(t)}\}$, the network topology at time slot $t $ can be abstracted as a WUG $\mathcal {G}(t)=(V(t), E(t)) $, where $V(t) $ denotes the vertex set, $E(t) $ denotes the edge set, and $\omega _{i}(t)$ denotes the vertex weight, and can be

\begin{equation*} \omega _{i}(t)=\sum _{j=1}^{|N_{i}^{s}(t)|}\beta _{s}\overline \delta _{i,j}(t)+ \sum _{k=1}^{|N_{i}^{a}(t)|}\beta _{a}\overline \delta _{i,j}(t),\tag{2}\end{equation*}

where $N_{i}^{s}(t)$ and $N_{i}^{a}(t)$ represent the symptomatic and asymptomatic neighbors of $v_{i}(t) $, respectively. Note that the parameter $\omega _{i}(t) $ quantifies the degree of risk. It is worth mentioning that the corresponding edge $e_{ij}(t)$ can only be inserted into $\mathcal {G}(t)$ if the social distance between the pair of vertices $v_{i}(t) $ and $v_{j}(t)$ in their contact is lower than a certain threshold. Note that the social distance threshold was initially three inches as declared by the World Health Organization (WHO), because the authors of [14] found that people who kept at least three inches of social distance between them were able to reduce the infection rate by approximately 82%. As a further result [1], the authors have demonstrated that two meters of social distance reduced infection rate by approximately 96%, since this social distance can prevent the transmission of droplets [14]. Hence here we use the latter metric as the threshold of social distance.

In a resource-constrained environment, we assume that the arrival process of medical resources obeys the Poisson distribution with arrival rate $\lambda $. Hence, we have to allocate these resources to those individuals at high-risk of being exposed in order to cut off the transmission paths, i.e. to remove the corresponding edges from $\mathcal {G}(t)$. Let $I(t)$, $N(t)$, $R(t)$, and $D(t)$ denote the number of infected individuals, all the individuals, all available resource blocks and the detected individuals, respectively. Here, we assume that a resource block can only be assigned to a single individual for detection. Therefore, the optimization problem is that of minimizing the infection rate (propagation speed), which can be formulated as

\begin{align*}&\min _{D} \sum \nolimits _{t=1}^{T}\dfrac {I(t)}{N(1) } \tag{3a}\\&s.t. C1: D(t)\leqslant {R(t)},\quad \forall t\in \mathcal {T}, \tag{3b}\end{align*}

where $(C1) $ indicates that the number of detected individuals should be no higher than that of the available resources. In graph theory, a vertex cover of $\mathcal {G}(t)$ is a subset of the vertex set $V_{c}(t)\subseteq V(t)$ so that for every edge, at least one of its endpoints belongs to $V_{c}(t)$. The MWVC is a vertex cover having the lowest possible number of vertices and sum weights. For the convenience of problem transformation, we turn the edge weights into negative values.

Proposition 1 (Detection Threshold): The MWVC of $\mathcal {G}(t)$ is the minimum number of vertices required for cuttikng off all the transmission paths (remove all edge) from $\mathcal {G}(t)$.

Proof: Considering the definition of MWVC, all edges can be omitted by removing the edges connected to $V_{c}(t) $ and meanwhile the $V_{c}(t) $ has the minimum size, which concludes the proposition 1.

Therefore, the problem (3) can be naturally transformed into a MWVC problem. It is worth mentioning that we consider the MWVC rather than MVC, because the former outperforms the latter when the size of minimum vertex cover exceeds the resources available. Next, we will investigate how to select higher-risk vertices from a MWVC.

## III. Adaptive Identification Scheme

In this section, we propose an adaptive identification scheme, which incrementally identifies the high-risk vertices instead of identifying all of them at once. More specifically, the adaptive identification process is divided into several rounds. In each round, our scheme can dynamically block the epidemic propagation based on a snapshot of the current network topology. Upon considering the case of $R(t)\leq V_{c}(t)$ at time slot $t$, selecting any $R(t)$ vertices from the set of $V_{c}(t) $ for detection will lead to different results. Given a limited budget, we should grant different priorities according to the associated hazard levels, which can be quantified by the vertex weight.

Since we have to dynamically find MWVC in the face of evolving network topologies obeying different graph structures, we first utilize the graph embedding process, in which each vertex can be represented by a $m$-dimensional vector paving the way for the learning phase. This is because reinforcement learning is more efficient when processing low-dimensional vectors than graphs. Similar to [11], we capitalize on the deep learning architecture termed as Structure2Vec [15] for graph embedding, which computes a $m$-dimensional feature embedding $\mu _{v_{i}}$ for each vertex $v_{i}$. Initially, we set $\mu _{v_{i}}(t)=0$ and the update strategy can be formulated as [11]

\begin{align*}&\hspace {-.7pc} \mu _{v_{i}}(t+1) =\text {relu}(\theta _{1}x_{v_{i}}+\theta _{2}\sum _{v_{j}\in NB_{i}(t)}\mu _{v_{j}}(t) \\&\qquad\qquad\qquad\qquad\qquad \displaystyle {+\,\theta _{3}\sum _{v_{j}\in NB_{i}(t)}\text {relu}(\theta _{4}\omega _{ij}(t))),} \tag{4}\end{align*}

where the neighbor set $NB_{i}(t)$ is equivalent to $N_{i}^{s}(t) \cup N_{i}^{a}(t)$, ’relu’ represents the rectified linear unit, i.e., $\text {relu}(\cdot)=\max (0,\cdot) $, $x_{v_{i}}$ is a binary variable, which indicates, whether the state is being selected or not, $\theta _{1}\sim \theta _{4} $ are model parameters, and $\omega _{ij}(t)$ represents the edge weight in graph embedding, which is determined by the structural similarity (structural distance) $f_{ij}(t)$, i.e., $\omega _{ij}(t)=e^{-f_{ij}(t)}$. Note that $f_{ij}(t) $ can be calculated by dynamic time warping (DTW) [15].

When the computation process of graph embedding is completed and the representation $h(S(t))$ of the selected vertex set $S(t)$ is obtained, we then define the evaluation function $\hat {Q}(h(S(t)),v_{i},\Theta)$, where $\Theta $ is the associated neural network parameter. When $\mu _{v_{i}}(T)$ obtained at the final slot, the pooled embedding of the entire graph $\mathcal {G}=\{ \mathcal {G}(t)\}_{t=1}^{T}$ can be represented as $\sum _{v_{i}\in S(T)}\mu _{v_{i}}(T)$. In this way, $\hat {Q}(h(S(t)),v_{i},\Theta)$ can be approximated by

\begin{align*} \hat {Q}(h(S(t)),v_{i},\Theta)=\theta _{5}^\top \text {relu}\left({\left[{\theta _{6}\sum _{v_{i}\in S(T)}\mu _{v_{i}}(T),\theta _{7}\mu _{v_{i}}(T)}\right]}\right), \\\tag{5}\end{align*}

where $[\cdot,\cdot] $ denotes the concatenation operator and $\Theta =\{\theta _{j}\} _{j=1}^{7}$.

Next, we will invoke reinforcement learning for determining the function $\hat {Q}(h(S(t)),v_{i},\Theta)$. In the neural network, the $n $-step fitted Q-Learning [11] is invoked to train $\Theta $. We define the states, actions and rewards in the reinforcement learning framework as follows:

• States: the selected vertices for detection at slot $t $, i.e., $S(t) $.
• Transition: the state variable $x_{v_{i}} $.
• Actions: push a new vertex into $S(t) $.
• Rewards: the reward function $r(S(t), v_{i}(t)) $ is defined as the change in the cost function after taking action $v_{i} $ and transitioning to a new state $S'(t) $, which can be expressed as
  \begin{equation*} r(S(t), v_{i}(t))=-1+\omega _{i}(t). \tag{6}\end{equation*}
  Note that we set a penalty of −1 for the increment in vertex number and $\omega _{i}(t) $ for the increment in weight to ensure that we can find a MWVC.

The training phase based on $n $-step fitted Q-learning is illustrated in Algorithm 1. Note that the termination criterion is whether the MWVC is achieved, i.e. whether all edges are covered, while the sum weights are minimum.

Algorithm 1 **Q-Learning Based Training for MWVC**
1: **Input:** Adjacency matrix of $\mathcal {G}$
2: **Output:** Parameter $\Theta $
3: Experience replay memory $\mathcal {M}$ is initialized to $N$
4: **for** episode $l=1:L$ **do**
5: Initialize the state $S(1) $ to empty set
6: **for** $t=1:T $ **do**
7: $v_{i}(t)= $
8: \begin{align*} \begin{cases} \text {randomly selection from } V\setminus S(t),& {\text {w.p.} \epsilon },\\ \arg \max _{v\in V\setminus S(t)}\hat {Q}(h(S(t)),v_{i},\Theta),& {\text {Otherwise}}, \end{cases}\end{align*}
9: Push $v_{i}(t) $ into $S(t+1)$, i.e., $S(t+1)=S(t)\cup v_{i}(t)$
10: **if** $t\geq n $ **then**
11: Push tuple $(S(t-n),v_{t-n},R_{t-n,t},S(t)) $ to $\mathcal {M}$
12: Randomly sample batch from $\mathcal {B}$
13: Update $\Theta $ by stochastic gradient descent to minimize the squared loss $(y-\hat {Q}(h(S(t)),v_{i},\Theta))^{2}$ for $\mathcal {B}$
14: **end if**
15: **end for**
16: **end for**

Note that the $n $-step Q-learning can handle the issue of delayed rewards during an episode by waiting $n $ steps before updating the parameters. This fits our scenario quite naturally, where the final objective value is only revealed after the addition of a series of vertices. In this way, the reward received so far can be used for estimating that in the future more accurately. Hence, the parameter $y $ in the squared loss function can be expressed as

\begin{align*} y\!=\!\sum _{k=1}^{n}r(S(t\!+\!k),v_{i}(t+k))\!+\!\gamma \max _{v'}\hat {Q}(h(S(t\!+\!n)),v',\Theta). \\\tag{7}\end{align*}

Furthermore, the fitted Q-iteration will rely on experience replay for updating the Q-function using a batch of samples instead of updating it sample-by-sample. In this process, the cumulative rewards $R_{t-n}$ can be represented by $R_{t-n,t}=\sum _{k=0}^{n-1}r(S(t-n),v_{i}(t-n))$. Based on the above discussion, our risk-aware adaptive identification (RAI) algorithm can be summarized in Algorithm 2.

Algorithm 2 **Risk-Aware Adaptive Identification (RAI)**
1: **Input:** Adjacency matrix of current snapshot of $\mathcal {G}$, i.e., $\mathcal {G}(t) $, available resource $R(t) $
2: **Output:** A set of vertices $V_{c}(t) $ at risk of being infected
3: Initialize the state $S(t)$ to empty set
4: Each vertex in $\mathcal {G}(t) $ is embeded into a $m$-dimensional vector using Eq. (4) and (5)
5: Search for the MWVC $S(t) $ using the Algorithm 1
6: **if** $|S(t)|> R(t) $ **then**
7: Select $R(t) $ vertices from $S(t) $ as $V_{c}(t) $ based on the ascending order of their weights
8: **else**
9: $D(t)\leftarrow S(t) $
10: **end if**

As shown in Fig. 1, when the furst stage of contact tracking is accomplished, graph embedding is performed to obtain the “node score” (green bars), which quantifies the degree of risk from the perspective of graph structure. In the final stage, reinforcement learning is invoked for solving the MWVC by considering both the node score and weight. The specific nodes associated with a high degree of risk are marked in red.

## IV. Simulation Results

In this section, we first evaluate our proposed scheme on the Erdos-Renyi social graph [16] relying on adjustable contact probability (edge insertion probability) and then on a pair of real-world datasets [12], [17]. The data in [12] collected from a primary school was used for evaluating our proposed scheme on a small-scale dynamic network and that in [17] collected from a museum was used for characterizing our proposed scheme on a large-scale dynamic network. In the dataset [17], 410 vertices are connected by 17,298 edges and the time span of 1 hour is discretized into 8 time slots. By contrast, in [12], 242 vertices are connected by 125,773 edges and the time span of 1 hour is discretized into 18 time slots.

In the learning phase, we set the batch size to 64, embedding dimension size to 64, the number of iterations to 5, $n $ to 5, $\epsilon $ to 0.05, training size to 10000, and the learning rate to 0.0001 based on [11]. For the SEIR model, we set the $\beta _{s} $ to 0.8, $\beta _{a} $ to 0.4, $\alpha _{s} $ to 0.7, and $\gamma $ to 0.3 based on the current data analysis about COVID-19 [4]. The initial number of randomly infected individuals is $0.2N $, where $N$ represents the number of all vertices in the initial stage. The arrival rate of resources ranges from 0 to 0.05 per second. Note that the vertices corresponding to the removed individuals will be removed from the current snapshot.

To comprehensively characterize our proposed scheme, we further compare RAI to four benchmarks: 1) degree centrality (to measure the risk of being infected by neighbors) selects $D(t) $ vertices with highest degree in the current snapshot [18], 2) betweenness centrality (to measure the risk of being infected on a large scale) can be calculated by

\begin{equation*} C_{B}(v)=\sum _{u\neq s \neq v \in V(t)}\dfrac {N_{s,u}(v)}{N_{s,u}}. \tag{8}\end{equation*}

where $N_{s,u}$ denotes the number of shortest paths connecting $s $ and $v $, and $N_{s,u}(v) $ denotes those shortest paths passing through $v $ [18], 3) closeness centrality (measure the risk of being infected on a small scale) can be calculated by

\begin{equation*} C_{c}(v)=\dfrac {|V(t)|-1}{\sum _{u \neq v \in V(t)}d_{u,v}}, \tag{9}\end{equation*}

where $d_{u,v}$ denotes the length of shortest paths connecting $u $ and $v$ [18], and 4) Q-learning based greedy algorithm for MVC (abbreviated as “GreedyMVC”) [11], which approximates the set of MVC nodes of the input graph by greedily selecting the uncovered edge having the maximum sum of degrees of its endpoints. Then we protect $k$ nodes from this unordered MVC set.

**FIGURE 2.**
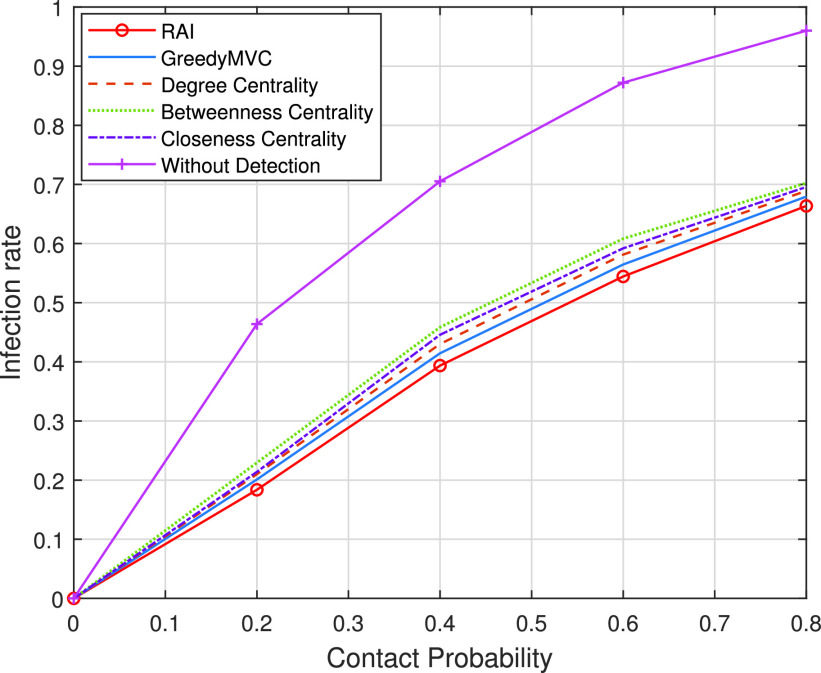
Infection rate versus the varying contact probability.

To verify the scalability and efficiency of our proposed scheme, we have conducted simulations relying on the classic Erdos-Renyi social graph, which is representative of most of the popular graph structures associated with varying contact probability. Fig. 2 shows the infection rate vs. contact probability, when the arrival rate of resources is set to 0.04 per second and the number of vertices is set to 100. The contact duration obeys the normal distribution associated with the expectation of 30s and standard deviation of 5s. The number of contacts obeys a Poisson distribution and the arrival rate is randomly chosen from $\{1,2,3\} $ per time slot. The time span of 1 hour is discretized into 10 time slots. We can observe that our proposed RAI always outperforms other methods upon increasing the contact probability. Compared to the scenario of “without detection”, our proposed scheme achieves approximately 43% infection rate reduction, when the contact probability is set to 0.8, which demonstrates the efficiency of early detection of highly suspected cases. Furthermore, since the degree and closeness centralities always select those specific vertices which are close to each other in the crowded parts of the graph, they exhibit a higher propagation rate than that of betweenness centrality (selecting the vertices which are in most of the multi-hop neighbor sets of other vertices) when the graph becomes dense, i.e., the number of edges becomes high.

**FIGURE 3.**
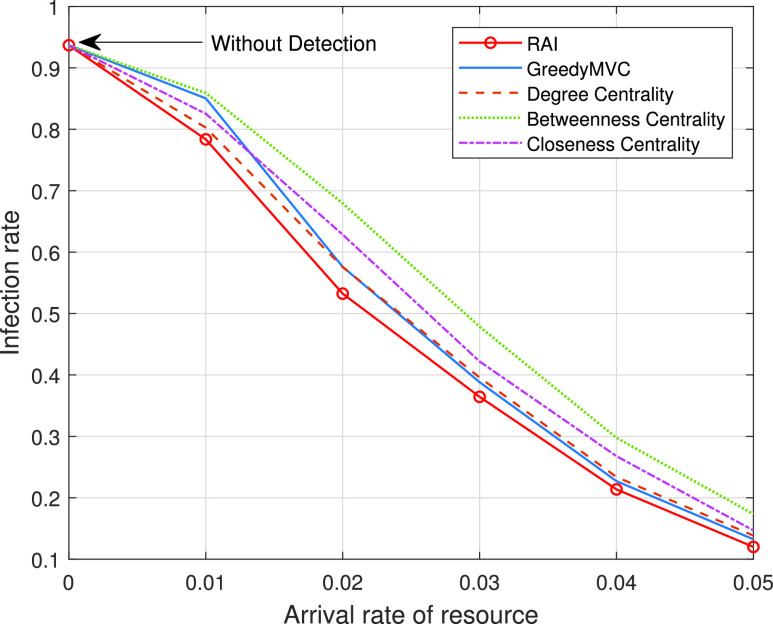
Infection rate versus the arrival rate of resource on dataset [12].

**FIGURE 4.**
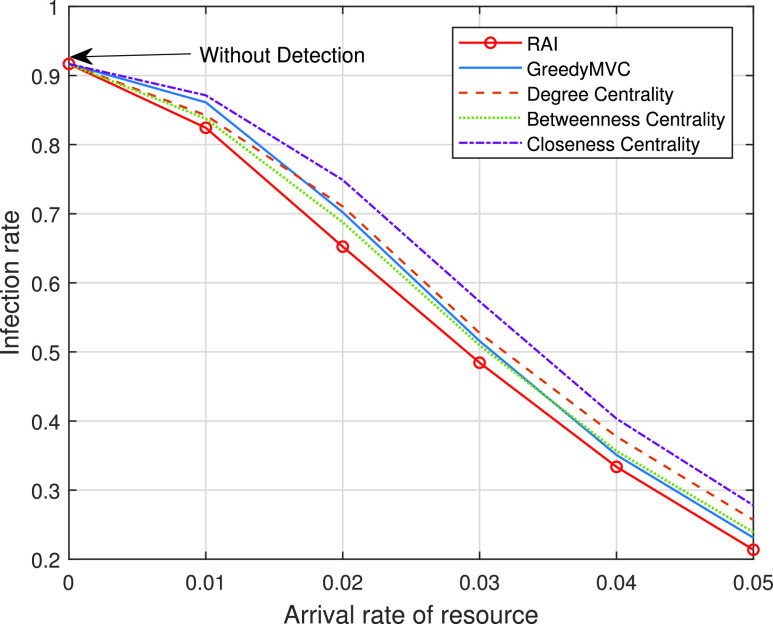
Infection rate versus the arrival rate of resource on dataset [17].

In Fig. 3 and Fig. 4, we study the impact of early detection based on two different real-world datasets. As clearly seen from these two figures, the proposed RAI scheme always outperforms the other methods upon increasing the arrival rate of resources for both datasets. In the small-scale scenario, the dominant form of infection is direct person-to-person transmission (one-hop transmission), while in the large-scale scenario, the cross infection (multi-hop transmission) becomes dominant. When the resources are very scarce, say for an arrival rate below 0.02 per second, the degree centrality method outperforms the “GreedyMVC”, because the degree centrality represents the degree of risk better within a limited budget. Upon increasing the arrival rate, the “GreedyMVC” performs better, because it can cut off more transmission paths within a certain budget. Note that the performance achieved by closeness centrality is better than that of betweenness centrality based on the dataset [12], but based worse on [17]. This is because the closeness centrality is efficient in small-scale scenarios (the selected vertices rapidly infect their one-hop neighbors) while the betweenness centrality is efficient in large-scale scenarios (the selected vertices can infect more multi-hop neighbors).

Finally, we discuss some characteristics inferred from the simulation and experimental results. Although the COVID-19 datasets are not available, Fig. 2 readily justifies that the proposed method outperforms the graph-theoretic methods for diverse graph structures. This is because the control efficiency is only related to the graph structures determined by the densities of the vertices and edges, plus the vertex weight. On one hand, the graph embedding is eminently suitable for extracting the centrality features of different graph structures. On the other hand, by solving the MWVC, the vertex weight is also considered. Since the traditional graph-theoretic based methods only consider the centrality feature, our proposed method would outperform the graph-theoretic based methods, regardless of the specific nature of the dataset.

Secondly, we justify the employment of reinforcement learning. Generally, traditional techniques of solving the graph optimization problem cam be classified into three main categories: exact algorithms, approximate algorithms and heuristic algorithms [11]. The exact algorithms perform well for small-scale scenarios, but their complexity tends to become prohibitive for large-scale scenarios. The approximate algorithms tend to have realistic complexity, but fail to provide sufficiently strong optimality guarantees. Finally, the heuristic algorithms tend to be efficient, but lack of theoretical guarantees. However, all three types of algorithms may only adapt to partial graph structures and thus their performance may be degraded in dynamically time-varying environments. Fortunately, this problem can be solved by reinforcement learning. It was demosntrated by extensive simulations in [11] that reinforcement learning based algorithms are capable of performing well in continuously envolving graph structures. Although different real datasets correspond to different scenarios, the graph structures inferred can typically be handled by relying on the Erdos-Renyi social graph. Futhermore, the reward function and other parameters are also influenced by the specific graph structures. As for the reward function, we carefully take into account the specific number of vertices and the vertex weight, which allows us to satisfy both the “minimum-weight” and “minimum set cover” conditions. Hence, the reward function used in the paper is suitable for the MWVC problem in the context of different graph structures. For other learning related parameters, the authors of [11] have indeed justified that this setting is suitable for most graph structures and thus we do not discuss this issue in detail. In conclusion, our proposed scheme exhibits excellent scalability and it is expected to perform well for diverse datasets, including COVID-19 datasets.

## V. Conclusion

In this paper, we have studied how to exploit the social relationships between mobile devices in SIoT to help control the infection rate by the early identification of suspected COVID-19 cases. Then, we transformed the optimization problem into a MWVC problem and proposed a RAI algorithm for solving this problem for a dynamic network topology. By relying on a pair of realistic datasets, we demonstrate that our scheme substantially reduces the epidemic infection rate compared to the benchmarks in both large-scale and small-scale scenarios. In conclusion, the proposed technique is eminently suitable for disease control and prevention by relying on the early identification of COVID-19 cases. At the time of writing no COVID-19 dataset is available concerning the accurate contact history of a crowd and their subsequent health conditions, but no doubt, real-life datasets will soon be available. This contribution may however assist both governments and other decision-making authorities in their decision making. In our future research we will use more data sources to verify and revise this early identification scheme at an increased accuracy.
